# Pigment Epithelium Macroadenoma Mimicking Iris or Ciliary Body Melanoma

**DOI:** 10.18502/jovr.v16i2.9100

**Published:** 2021-04-29

**Authors:** Sara Sánchez-Tabernero, Ciro García-Alvarez, Elena García-Lagarto, Maria A Saornil

**Affiliations:** ^1^Department of Ophthalmology, Anterior Segment Service, Moorfields Eye Hospital, London, UK; ^2^Servicio de Oftalmología, Unidad de Tumores Intraoculares del Adulto, Hospital Clínico Universitario de Valladolid, Spain; ^3^Unidad de Patología, Hospial Clínico Universitario de Valladolid, Spain

##  PRESENTATION

A 66-year-old man presented to the Intraocular Tumor Unit at Hospital Clínico Universitario of Valladolid, Spain, with a one-year history of gradual vision loss in the left eye. The patient had previously undergone cataract surgery. Examination revealed a mass arising from the iris, invading the iridocorneal angle and ciliary body, and displacing the intraocular lens posteriorly. The dimensions were 11.51 × 11.39 × 7.53 mm, as measured under ultrasound biomicroscopy. The mass was hyperintense on T1- and hypointense on T2-weighed magnetic resonance images. This is the most frequent pattern described in ciliary pigment epithelium adenomas, although hyperintensity on both T1- and T2-weighted images has also been reported.^[1]^ Enucleation was performed because of suspected iris melanoma. Histopathology demonstrated nests and cords of pigmented epithelial cells with an adenoid pattern, consistent with previous studies.^[1,2]^ Atypia, mitotic figures, or infiltrative features were not observed. Histopathology was diagnostic of macroadenoma of iris pigment epithelium, although a ciliary body origin could not be excluded.

**Figure 1:**
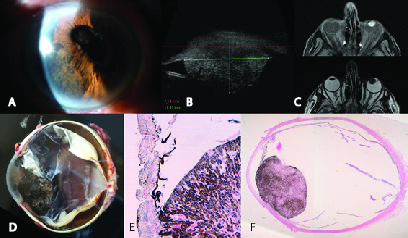
(A) Slit-lamp biomicroscopy showing an iris mass. (B) Ultrasound biomicroscopy. (C) Mass on T1- and T2-weighed magnetic resonance images. (D) Enucleated eye. (E&F) Hematoxylin and eosin stain, 4× and low-power magnification.

##  DISCUSSION

Histopathology demonstrated nests and cords of pigmented epithelial cells with an adenoid pattern, consistent with previous studies.^[1,2]^ Atypia, mitotic figures, or infiltrative features were not observed.

Histopathology was diagnostic of macroadenoma of iris pigment epithelium, although a ciliary body origin could not be excluded.

##  Financial Support and Sponsorship

Nil.

##  Conflicts of Interest

The authors declare no interests.
